# Odontogenic Cutaneous Sinus Tract in a 10-Year-Old Girl: A Case Report of a Rare Entity

**DOI:** 10.7759/cureus.39156

**Published:** 2023-05-17

**Authors:** Shojiro Hanaki, Shuichi Katayama, Yoshihisa Watanabe

**Affiliations:** 1 Division of Pediatric Surgery, Department of General Surgery, Kurashiki Central Hospital, Kurashiki, JPN

**Keywords:** odontogenic sinus tract, cervical masses, dental caries, pediatric, children

## Abstract

Odontogenic cutaneous sinus tract (OCST) is defined as pulp necrosis caused by dental caries or trauma that forms a fistula on the body surface as a drainage channel for the infected pulp. OCST can be difficult to diagnose because subjective symptoms, such as pain in the affected tooth, may be minimal. In addition, lesions in the cervical region are extremely rare. In this report, we discuss the case of a 10-year-old girl who presented with inflammation, edema, and purulent exudation on the right neck. Her symptoms resembled those of lateral cervical cysts and fistulas. However, upon evaluation, she was diagnosed with OCST. Although OCST is an important differential diagnosis for head and neck lesions, it is often overlooked. OCST should be considered in the differential diagnosis of neck masses and fistulas.

## Introduction

Odontogenic cutaneous sinus tract (OCST) is defined as pulp necrosis caused by dental caries or trauma that forms a fistula on the body surface as a drainage channel for the infected pulp [1]. The diagnosis of OCST can be difficult because subjective symptoms, such as pain in the affected tooth, may be minimal [2]. Despite being an important differential diagnosis for head and neck lesions, OCST is often overlooked. This report presents the case of a child with cervical symptoms, which are extremely rare. This report is intended to raise awareness among clinicians who treat pediatric patients, such as pediatricians and pediatric surgeons, of the possible dental origin of cervical lesions and the importance of considering an OCST in the differential diagnosis of head and neck lesions.

## Case presentation

**Figure 1:**
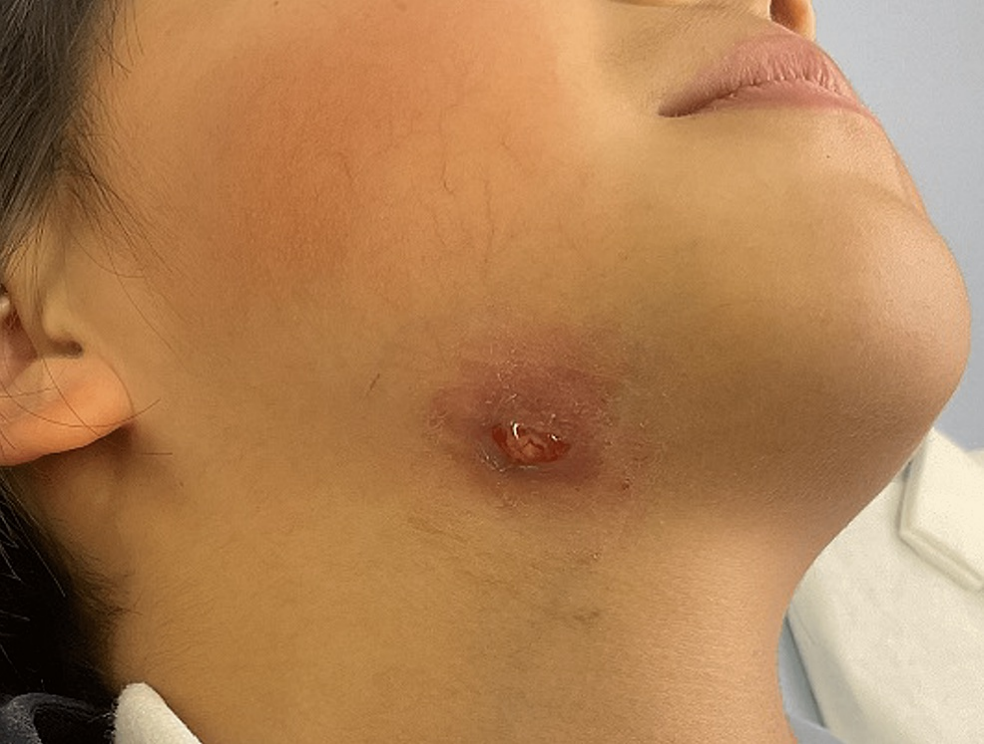
Preoperative appearance Preoperative photograph of the extraoral appearance from the right mandible to the neck, showing inflammation, edema, and purulent exudation.

A 10-year-old girl presented to our hospital with inflammation, edema, and purulent exudation on the right neck. One year earlier, she had been treated with antibiotics by her primary care physician for a mass on the right neck. She was initially diagnosed with lymphadenitis. However, the mass recurred periodically, requiring incision and drainage. A lateral cervical cyst or fistula was suspected, and the patient was referred to our hospital for workup. On examination, inflammation, edema, and purulent exudation were observed from the right neck, with mild tenderness on palpation (Figure 1).

**Figure 2:**
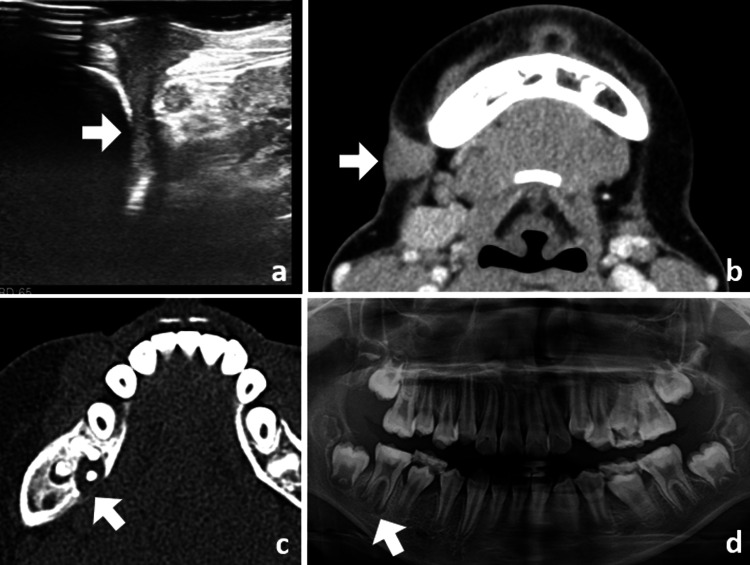
Preoperative images (a) Ultrasonography showing a fistula extending from the skin to the mandible. (b) An axial CT scan showing fistula formation and associated inflammation. (c) A focal cortical bone defect in the mandibular right first molar, and interruption of the lingual cortex and periosteal reaction. (d) A panoramic radiograph showing an enlarged periodontal ligament and apical periodontitis in the mandibular right first molar, with osteosclerosis around it. (All indicated by arrows)

Ultrasonography revealed a fistula extending from the body surface to the mandible (Figure 2a), and the CT scan showed fistula formation and associated inflammation (Figure 2b), a focal cortical bone defect in the mandibular right first molar, and interruption of the lingual cortex and periosteal reaction (Figure 2c). A panoramic radiograph showed an enlarged periodontal ligament and apical periodontitis in the mandibular right first molar, with osteosclerosis around it (Figure 2d).

**Figure 3:**
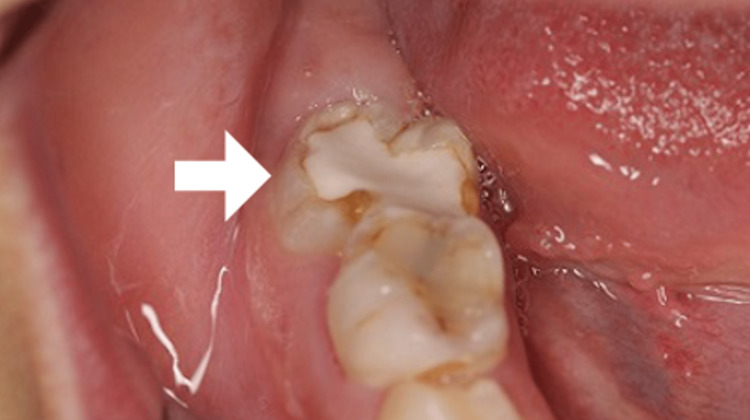
Intraoral photo Intraoral photo showing the recurrent caries in the crown restoration of the mandibular right first molar (the arrow).

Her dental history revealed that a carious tooth in the same area had been restored with a crown several years earlier. Suspecting odontogenic disease, she was referred to a dentist, who found recurrent caries in the crown restoration of the mandibular right first molar, accompanied by apical periodontitis (Figure 3).

**Figure 4:**
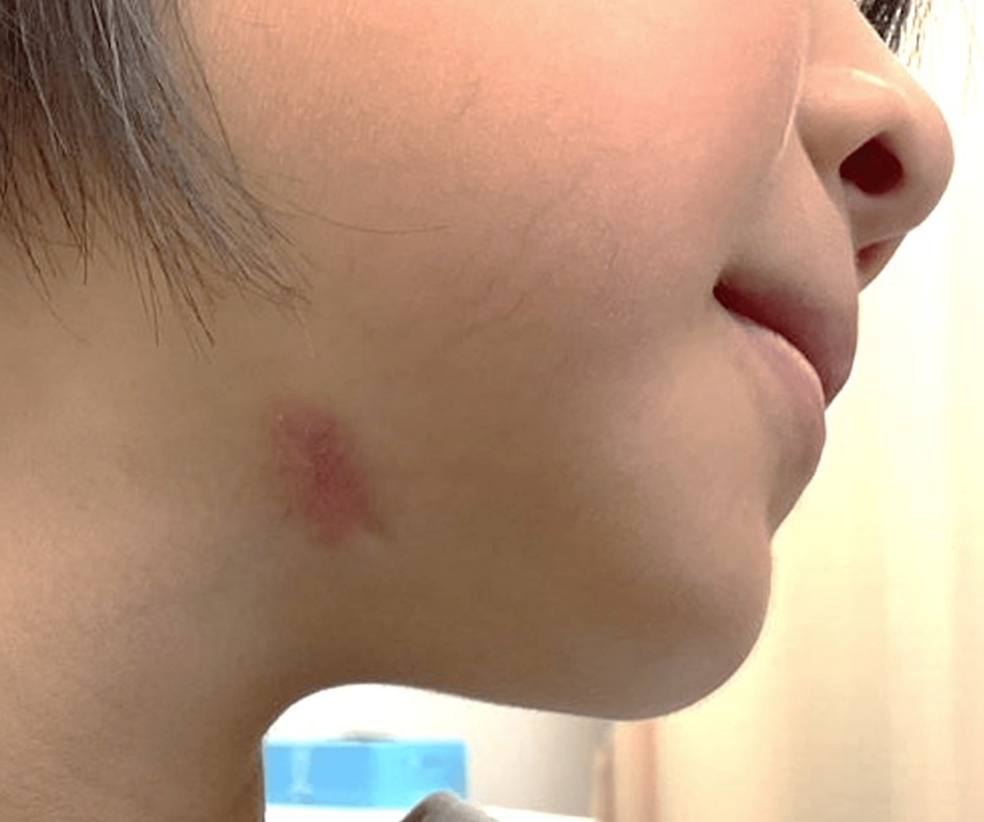
Postoperative appearance Photograph showing the postoperative appearance three months after surgery.

On the basis of these findings, a diagnosis of OCST due to apical periodontitis of the mandibular right first molar was made, and the tooth was extracted and the granulation tissue was removed. Symptoms improved rapidly after surgery, with no recurrence of symptoms 10 months after surgery (Figure 4).

## Discussion

The main cause of OCST is apical periodontitis, a necrosis of the dental pulp caused by dental caries or trauma [3,4]. OCST can be caused by other factors besides apical periodontitis, such as impacted wisdom teeth, osteomyelitis, and advanced periodontal disease [5]. Fistulas that form in the oral cavity, especially in the gingiva, are called internal dental fistulas, while those that form on the facial skin are called OCST [6]. The prevalence of internal dental fistulas has been reported to be about one in five teeth with periodontitis, while OCST is relatively rare [7]. OCST is often described in the dental literature but is difficult to diagnose because patients may present with minimal subjective symptoms, such as pain in the affected tooth. As a result, OCST patients are treated by non-dental specialists such as dermatologists, plastic surgeons, or, in the case of pediatric patients, pediatricians, which can lead to delayed diagnosis and potentially inappropriate treatment [8,9].

The primary site of OCST is the mandibular molar, which has a high incidence of dental caries. Mandibular canines are the second most common cause of pyogenic pulpitis because their roots are long and do not exfoliate until old age [10,11]. OCST occurs in all age groups, but in children, it is more common in teenagers and less common in children under 10 years of age [12,13]. The buccal space is the most common site of the fistula, followed by the mental, masseter, and submandibular space [9]. Variation in the fistula site is related to the location of the causative tooth, the length of the root, the location and thickness of the surrounding muscle attachments, the density and sparsity of the subcutaneous tissue, the virulence of the infection, the thickness of the cortical bone, and the effect of gravity [3,7]. In a previous report, the site of fistula formation has been correlated with the causative tooth, with mandibular molars often forming fistulas in the buccal space, mandibular premolars in the molar space, and maxillary premolars in the nasal wing base [5]. Lesions close to the neck are extremely rare.

Panoramic radiographs are an effective diagnostic tool for identifying OCST by demonstrating the radiolucency of the affected teeth [4]. However, panoramic radiographs are often taken by dentists and, moreover, are not usually taken unless the odontogenic disease is suspected. In this case, a panoramic radiograph was taken after OCST was suspected on a CT scan. A CT scan is particularly useful in the diagnosis of OCST because it can show characteristic features such as cord-like structures in the surrounding subcutaneous soft tissue and fractures of the cortical bone, and it is also useful in differentiating OCST from other superficial skin diseases [14]. In this case, we also suspected odontogenic disease and decided to perform a CT scan because ultrasonography findings showed a fistula leading to the mandible. Ultrasonography is a minimally invasive, easily accessible imaging modality that is particularly useful in pediatric patients.

Once OCST is confirmed, the first step is to treat the causative tooth. This may include endodontic therapy or surgical root canal therapy and apicoectomy for restorable teeth, or extraction of nonvital teeth to eradicate infection. While the standard protocol involves resection of the fistula in conjunction with the treatment of the causative tooth, even when the causative tooth is treated alone, the fistula often resolves within two weeks, resulting in a good outcome [3,15]. OCST is cured by dental treatment only and does not require special antibiotic treatment.

This case resembles a lateral cervical cyst and fistula both in appearance and history [16]. Lateral cervical cysts are known to arise from remnants of the second branchial arch and those that communicate with the skin are called lateral cervical fistulas [17]. The typical treatment course for lateral cervical cysts and fistulas is the surgical removal of both the cyst and the fistula [18]. If this case had been misdiagnosed as a lateral cervical cyst and fistula and only the fistula had been resected without addressing the underlying dental pathology, the patient’s condition would not have improved and the treatment course would have been prolonged. In the case of facial and neck lesions with secretions, considering the possibility of a dental origin is imperative for the clinician to make an accurate diagnosis and a treatment plan. OCST is recognized as a major cause of delayed diagnosis and treatment because patients often do not report oral symptoms despite the presence of a dental infection [19]. This is thought to be because the abscess is drained through the skin, relieving pain and swelling [3]. A history of recurrence and a previous need for drainage are very important clues to suspect OCST. To identify odontogenic disease, it is important to obtain a detailed history, including information on dental caries, periodontal disease, previous dental trauma, and endodontic therapy. In addition, performing an oral examination and identifying teeth with severe caries or crowns may suggest that the lesions are of dental origin [20]. Clinicians who treat pediatric patients, such as pediatricians and pediatric surgeons, should be aware of the possibility of odontogenic disease without performing an oral examination, and obtaining a verbal history may allow early detection and intervention.

## Conclusions

This case report highlights the importance of considering OCST as a possible differential diagnosis for cervical lesions in pediatric patients. Awareness among clinicians treating pediatric patients is necessary for the early diagnosis and appropriate management of OCST.
